# Differential executive functioning in the topology of Spirit possession or dissociative disorders: an explorative cultural study

**DOI:** 10.1186/s12888-019-2358-2

**Published:** 2019-12-02

**Authors:** Samir Al-Adawi, Yahya Al-Kalbani, Sathiya Murthi Panchatcharam, Matlooba Ayoub Al-Zadjali, Sara S. Al-Adawi, Musthafa M. Essa, M. Walid Qoronfleh

**Affiliations:** 1 Department of Behavioral Sciences, College of Medicine and Health Sciences, Sultan Qaboos University, Muscat, Sultanate of Oman; 2 Studies and Research, Oman Medical Specialty Board, Muscat, Oman; 3 Ministry of Health, Directorate of Non-Communicable Diseases, Muscat, Sultanate of Oman; 4 Oman Medical Specialty Board, Muscat, Oman; 5 Department of Food Science and Nutrition, College of Agricultural and Marine Sciences, Sultan Qaboos University, Muscat, Oman; 6 Research & Policy Department, World Innovation Summit for Health (WISH), Qatar Foundation, P.O. Box 5825, Doha, Qatar

**Keywords:** Intermittent dissociative phenomenon, Transitory dissociative phenomenon, Spirit possession, Executive functioning, Oman

## Abstract

**Background:**

In Oman, anecdotal and impressionistic observation have helped parse and categorize various manifestations of spirit possession into two broad and distinct categories: intermittent dissociative phenomenon and transitory dissociative phenomenon. The primary aim of the present study was to compare the performance of participants on neuropsychological tests among different grades of possession. Other correlates were also sought.

**Methods:**

Assessment criteria for the two groups included measures examining executive functioning: controlled oral word association test *Verbal Fluency*, *Wisconsin Card Sorting Test* (Perseverative error and the number of categories achieved)*, Trail Making Test* and the *Tower of London Test (*number of correctly solved problems*)*. Sociodemographic variables and the history of trauma were also sought.

**Result:**

Among 84 participants, one third of them presented the intermittent possession type and two thirds, the transitory possession type. Their mean age was 34.17 ± 11.82 and 56% of them were female. Nearly 35% of them endorsed a history of a traumatic experience. Both the multivariate models showed statistical significance (F (5, 78) = 5.57, *p* < 0.001, R^2^ = 0.22), F (5, 78) = 11.38, *p* < 0.001, *R*^2^ = 0.39) with an independent predictor of intermittent dissociative phenomenon (β = − 3.408, *p* < 0.001), (β = 63.88, *p* < 0.001) for *Verbal Fluency* and *Trail Making Test*, respectively. The history of the traumatic event was also statistically significant with the results of the *Trail Making Test* (β = − 26.01, *p* < 0.041. Furthermore, the subtype of Pathogenic Possession turned out to be an independent predictor across all models: *Wisconsin Card Sorting Test* perseverative error, Wisconsin card sorting test categories achieved and the number of problems solved in the *Tower of London Test* (OR = 3.70, 95% C.I. 2.97–4.61; *p* < 0.001), (OR = 0.57, 95% C.I.0.39–0.84; *p* = 0.004) and (OR = 0.80, 95% C.I. 0.65–0.99; *p* < 0.037) respectively.

**Conclusions:**

This study suggests that typology of spirit possession found in Oman tends to differ on indices of executive function. Those with ‘diagnosis’ of intermittent possession showed impairment in many indices of executive functioning. Despite its wide prevalence, spirit possession has not been examined in terms of its neuropsychological functioning. We believe that this study will be instrumental in laying the groundwork for a more robust methodology.

## Background

Dissociative phenomena and the concept that spirits may impersonate or possess human beings are found across 90% of the world’s population [1]. The most vivid illustration of these beliefs manifests as the phenomenon of spirit possession in which a person’s behavior is thought to be controlled by an anthropomorphic being that has entered a human being’s body (cacodemonomania) [2].

Early explorers described spirit possession using derogatory terms indicating it to be a remnant of primitive mentality [3]. Various contesting paradigms ranging from anthropology to epistemic premises have dictated discourse on these dissociative phenomena [1, 4, 5]. A number of authors subsequently started to describe spirit possession according to the prevailing social scientific paradigm. In the instance of the functionalist paradigm, Lewis [5] suggested spirit possession as a mechanism to channelize social discontent in society into a socially acceptable manner. Thus, spirit possession was deemed to be more common among marginalized members of society [6]. Believed to be inhabited by supernatural forces, the marginalized member of the society may regain a new status among society. Psychoanalytic perspectives depict spirit possession as a form of socioculturally sanctioned ‘pathology’ stemming from repressed Oedipal desires in the unconscious [7]. Outside the realm of ethnography, there are ample clinical studies of dissociative phenomena. Jean Etienne Esquirol [8] had initially suggested dissociative phenomena as a ‘disease’ but later dissociative and psychotic-like experiences were lumped under the label of dissociative identity disorder. According to [9], “the current psychiatric paradigm treats dissociation as a pseudo-adaptive, functional neurological response to the experience of acute stress and trauma” (p. 133).

Along with delineation of spirit possession using different schools of thought, complexity and the cosmology of spirit possession have been divided into two broad types. As suggested by Lewis [5]: ‘central’ versus ‘peripheral’ possessions. According to Halloy & Naumescu [10], “Central possession is an elective, socially valued possession, since it embodies beliefs and values of the dominant classes. The peripheral possession defines marginalized groups, especially women, characterized by their relation to malicious spirits, carriers of affliction and amorality” (p. 159).

Another influential figure in the anthropology of spirit possession, Bourguignon [1] has categorized the various types of possession into two broad types. The first one is characterized by trance. During the trance state, individuals experience an altered state of consciousness or bodily changes, but general society does not necessarily attribute this altered state to an ‘invading spirit’. In the second type of spirit possession, the afflicted individual presents an altered state of consciousness which the society directly attributes to an invading spirit. Thus, the possessed identity is replaced by another.

Cohen [4] has suggested two types of possession: ‘pathogenic possession’ and ‘executive possession’. Executive possession implies that the spirit has replaced the identity of the individual turning them into a different self for the duration of possession. For example, an individual may accrue power to read the future to combat impending danger. During this state, ‘selfhood’, as defined by western psychology, is transformed or replaced. Executive possession reflects the concept of adorcism which entails curative possessions or mechanisms to placate spiritual entities in a possessed person [11]. In pathogenic possession, the spirit causes illness in and misfortune for the affected individual. As often is the case, the presence of such a pathogenic spirit drives the family of afflicted individuals to resort to exorcism. According to Cohen, “a person may be pathogenically possessed by a particular spirit at one moment, and non-pathologically possessed by the spirit at another, depending on “whether the perceptual and conceptual inputs match the conditions that activate contamination systems or person-identity systems in cognition” (p. 120).

Per local Omani vernacular, there are two grades of spirit possession [3] which we have operationalized as ‘intermittent dissociative phenomenon’ and ‘transitory dissociative phenomenon’. In the intermittent dissociative phenomenon, the ‘possessed’ individual appears to have been ‘taken over’ by a spirit or other supernatural forces continuously, though the expression of possession might wax and wane. According to prevailing sociocultural beliefs, the non-being (a spirit or jinn) can manifest as a benevolent or malevolent force. In intermittent dissociative phenomenon, the incarnated is taken over by the malevolent force [3]. The intermittent dissociative phenomenon appears to always be malevolent. The concept of intermittent dissociative phenomenon also mirrors the description of pathogenic possession by Cohen [4] and resonates with peripheral possession detailed by Lewis [5]. When viewed from biomedical perspectives, the condition of the possessed individual manifests as something akin to the pathological type along with dissociative identity disorder or demonic possession [3].

The second type of spirit possession is that which is transitory in nature whereby the individual is possessed for a short duration, often associated with particularly stressful events.

Some of the spirits involved in the transitory dissociative phenomenon are believed to ‘influence’ the individual to accrue adaptive responses when he or she is confronted with the likelihood of misfortune. In the transitory dissociative phenomenon, the relationship between the spirit and the human are often symbiotic: the individual and the spirit have a reciprocal relationship of benevolence [12]. Per Euro-American psychiatric terminology, the transitory dissociative phenomenon might appear to be pathological but falls under “normal part of a broadly accepted cultural practice’ [13].

While literature taking an ethnographical and phenomenological approach to spirit possession is replete, there is a dearth of studies examining neuropsychological functioning in people with possession. Applying neuropsychological tools among people with dissociative phenomenon has “potential to explore the way that sociocultural systems structure not only the developmental experiences of individuals, but also the content and process of experience, thought, and behavior in everyday life in ways that have a recognizable trace at the neural level ([9], p.130).’

To our knowledge, there is a dearth of studies on cultural neurosciences among grades of spirit possession in Arab/Islamic societies. In order to fill this gap in existing literature, the aim of the present study was to compare the neuropsychological profile of Omanis with intermittent dissociative phenomenon vs. transitory dissociative phenomenon. The dissociative phenomenon has been proposed to be precipitated by psychological trauma [14]. This ‘trauma hypothesis’ has also been noted in non-western populations [15, 16]. A related aim of this study is to explore this hypothesis.

## Methods

### Setting

With a population of approximately 3 million, Oman has a loosely-systematized centralized healthcare system [17]. Mental health services are provided in primary healthcare centres, regional hospitals, and at two tertiary care hospitals for mental illness. Under the umbrella of the Ministry of Health, Oman provides universal access to free healthcare for all its citizens for both physical and mental health. This investigation was able to utilize the existing compartmentalized healthcare system in Oman to recruit participants for this study.

### Identification of Spirit possessed

A previous study in Oman has indicated that there is a widely held view in traditional societies that some people are relatively more prone to be taken over by a spirit, power, deity or other entity [3]. Those with such idioms of distress were invited for the present study. During the study period (from October 1, 2016 to March 31, 2017), 105 patients were noted to attribute their presenting signs and symptoms to spirit possession. Those consenting to participate in this study were subjected to a semi-structured interview using the style and format of the Composite International Diagnostic Interview (CIDI) to solicit the presence of Dissociative Identity Disorder, Dissociative amnesia or Depersonalization/derealization disorder according to the definitions and criteria of the *International Statistical Classification of Diseases and Related Health Problems* (ICD-10) [18] and *Diagnostic and Statistical Manual of Mental Disorders* (DSM [19];). Exclusion criteria entailed that the altered state of consciousness should not have been derived from a physical disorder or impaired sensorium as in the case of psychotic disorder, including schizophrenia or related disorders (F20-F29), or mood [affective] disorders with hallucinations or delusions (F30-F39). Participants indicating the presence of illicit drugs in their system during the routine urine drug screening were also excluded from the study. Similarly, participants currently on psychiatric medications were also excluded from the study. Thus, all participants were drug naive.

Participants who fulfilled the study criteria were interviewed using an explanatory model developed elsewhere [20, 21]. In brief, explanatory models gauged non-medical “attributions of a specific episode of illness that are held by patients, their family or practitioners. Predominantly culturally shaped, these models project personal and social meaning on the illness experience” (p. 175) [22]. The interview tackled questions revealing whether the participants indicated intermittent dissociative phenomenon or transitory dissociative grade of possession. More specifically, the open-ended interview briefly covered questions of (i) perceived factors that triggered the dissociation, (ii) frequency of the episodes of dissociation (iii) duration of episodes of dissociation and (iv) manifestation of the dissociation itself (e.g., malevolent vs benevolent).

Out of 105 subjects, 29 fulfilled the present requirement of the intermittent dissociative phenomenon and 55 fulfilled requirements of the transitory dissociative phenomenon, amounting to a final total of 84 participants.

### Traumatic experience

*The Trauma History Questionnaire* [23, 24] is a 24-item measure gauging different aspects of psychological trauma including crime, general disaster, and sexual and physical assault. The instrument required the participant to answer ‘yes’ or ‘no’. If they answered ‘yes’, the participants were asked to provide further information on the frequency and the age at the time of the event. There are many scoring systems for this scale [24]. For the present purpose, the scores were transformed into binary variables, i.e. ‘present’ or ‘not present’. For brevity, the present study simply reported ‘Yes’ or ‘No’.

### Neuropsychological assessment

With their consent, Omani nationals who fulfilled the criteria of possession syndrome were invited to take the neuropsychological assessments described below. All participants were given in writing that their data would be confidential, their participation in the study was completely voluntary, and that they could withdraw from the study at any time. In order to honour the beneficence principle, participants having significant neuropsychological impairment were given the option to repeat the assessment in order to rule out whether their neuropsychological functioning has reached a plateau or continued to deteriorate.

The neuropsychological evaluation was conducted at the Department of Behavioral Medicine and Psychiatry, Sultan Qaboos University. This particular unit is the only one in the country equipped with a neuropsychology laboratory.

Four neuropsychological measures were operationalized to index executive functioning as reported in the literature [25, 26].

(i). In the ***Controlled Oral Word Association Test****, or* ***Verbal Fluency Tes****t* [26], interviewees were asked to verbally improvise as many different words as possible that began with each of three specific letters relevant to Arabic Speakers as described elsewhere [27]. On this scale, lower scores denoted a higher propensity to neuropsychological dysfunction. Functional magnetic resonance imaging (fMRI), positron emission tomography (PET) scan and Single-photon emission computed tomography (SPECT) have often been employed to tap into brain activity or cerebral blood flow associated with performance on neuropsychological tests. Using such functional brain scanning tools, Price [28] has suggested that verbal fluency is associated with increased blood flow in the brain regions often associated with executive functioning including the dorsolateral prefrontal cortex and its reciprocal projections in other regions.
(ii). In the ***Wisconsin Card Sorting Test*** [29], the participants are given a set of cards which vary independently along three dimensions (color, shape and number of symbols) and are asked to sort these. They are not told which dimension should direct their categorisation but are told what is ‘right’ or ‘wrong’ after they have placed each card. Once they are consistently sorting according to one of the three dimensions, the ‘rule’ is switched so that the respondent now says ‘wrong’ to previously correct responses. Subjects must not only determine the three dimensions by which it is possible to sort, but they must also determine which one is correct at any time, maintain the rule over successive trials, and determine the new correct dimension on the basis of feedback after it is changed by the experimenter [30]. In the present context, two indices were recorded: the number of categories cleared and the number of perseverative errors. A perseverative error is defined as a failure to change the sorting strategy even after receiving negative feedback. On this scale, higher scores denoted a higher neuropsychological impairment. In a study using in vivo functional neuroimaging Bohon, Weinbach & Lock [31] have indicated that performance on the *Wisconsin Card Sorting Test* was associated with an increase in metabolic activity in right frontal pole, inferior frontal gyrus and middle frontal gyrus.
(iii). Scoring for the ***Trial Making Test*** [32] was derived from two versions of the test. The first required the examinee to draw a line connecting the numbers 1 through 25 in sequential order, each shown in a plain black circle. In the second version, the examinee drew a line to connect the numbers 1 through 12 and the letters A through L in alternating sequence, beginning with 1 and drawing a line to A, then 2, then B, and so on until all the numbers and letters are connected. In the present context, total times for both versions of the test were scored in seconds. In this scale, higher scores denoted propensity towards cognitive impairment. Zakzanis, Mraz & Graham [33] have reported greater brain activity in the dorsolateral and medial frontal to be associated with the performance on the *Trial Making Test*.
(iv). In the ***Tower of London Test*** [34] subjects were asked to rearrange differently-coloured beads on three upright pegs to match a model presented by the assessor, within a fixed number of single moves, and with a gradually increasing need for planning. For the present purpose, the focus was on the number of correctly solved problems with a maximum correct score of 12 as described elsewhere [27]. On this scale, lower scores denoted higher neuropsychological dysfunction. [35] have reported regional cerebral blood flow was significantly increased in the left pre-frontal cortex during the *Tower of London Test*.

### Statistical analysis

The collected data were analyzed using SPSS Statistics 25.0 (IBM Corp. Released in 2017. IBM SPSS Statistics for Windows, Version 25.0. Armonk, NY: IBM Corp.). Categorized variables were presented as frequency and percentages. Continuous outcome variables met approximate normal distributions and were analysed using linear regression and count data were analysed using the Generalized Linear Model (GLM) with poisson loglinear distribution. A *p*-value <0.05 was considered statistically significant.

### Ethical consideration

This study was approved by the Institutional Review Board, Research and Ethical Committee of the College of Medicine & Health Sciences, Sultan Qaboos University - SQU (EC 55/14/MREC #907). Participants were requested to provide written informed consent and study procedures were carried out in accordance with the Code of Ethics of the World Medical Association (Declaration of Helsinki) for human experiments.

## Results

A total of 84 subjects were studied. Their mean age was at 34.17 ± 11.82 and 56% of them were female. Approximately 65% of participants had less than university education while approximately 35% of them had acquired university education. Nearly 35% of them had a previous history of traumatic experience. Among the participants, about one third of them were marked with intermittent possession type and the rest of them were marked with the transitory dissociative phenomenon.

In terms of gender composition, among the 29 participants with intermittent possession, 22 were females. Among 55 indicating the transitory dissociative phenomenon, 25 were females while 30 were males.

Regarding the participant’s history of traumatic experiences, as shown in Tables 1, 29 participants fulfilled the criteria of intermittent possession while the rest (*n* = 55) belonged to the transitory dissociative phenomenon. Among the 29 with intermittent dissociative phenomena, 5 endorsed a history of trauma. Among 55 with ttransitory dissociative phenomenon, 24 endorsed history of trauma. There was an association observed between the topology of dissociative phenomena and traumatic experienced, which was statistically significant (*p* = 0.017). Among those that had experienced trauma, 83% presented currently defined transitory dissociative phenomenon. The indication of history of a traumatic event was also statistically significantly associated with the *Trail Making Test* (β = − 26.01, *p* < 0.041) and these participants showed more statistically significant preservative errors in *Wisconsin Card Sorting Test* (OR = 0.81, *p* < 0.020).

**Table 1:** The association between *Verbal Fluency* and *Trail Making Test*, subtypes of possession and socio-demographics (gender age and education and risk factors history of trauma)

Variables	n	Verbal Fluency				Trail Making Test			
		Unadjusted		Adjusted		Unadjusted		Adjusted	
		Mean (sd)	*p*-Value	β	*p*-Value	Mean (sd)	*p-*Value	β	*p*-Value
Gender									
Male	37	14.86 (2.98)	0.112	0.332	0.471	101.89 (2.46)	0.002	−24.03	0.050
Female	47	13.72 (3.42)				140.26 (78.72)			
Age									
≤ 30 years	44	13.80 (3.83)	0.198	−0.898	0.246	145.73 (79.53)	<0.001	−14.60	0.270
≥ 30 years	40	14.70 (2.47)				98.75 (17.82)			
Subtype									
Intermittent dissociative phenomenon	29	12.10 (3.03)	<0.001	−3.725	<0.001	175.76 (83.77)	<0.001	63.88	<0.001
Transitory dissociative phenomenon	55	15.35 (2.82)				95.73 (15.20)			
Education									
University	29	15.48 (2.98)	0.010	−0.008	0.993	94.69 (13.25)	<0.001	12.01	0.407
Less than University	55	13.56 (3.24)				138.47 (73.19)			
History of traumatic experience									
Yes	29	15.41 (3.58)	0.015	0.939	0.203	98.31 (18.41)	0.001	−260.01	0.041
No	55	13.60 (2.93)				136.56 (73.70)			

Table 1 describes the associations of *Verbal Fluency* and *Trail Making Test* with the factors using univariate and multivariate regression. It indicates that most of the factors showed statistical significance for the verbal fluency and trail making in the univariate model, except gender and age for the verbal fluency. Both the multivariate models showed statistical significance (F (5, 78) = 5.57, *p* < 0.001, *R*^2^ = 0.22), F (5, 78) = 11.38, *p* < 0.001, *R*^2^ = 0.39) with an independent predictor of intermittent dissociative phenomenon (β = − 3.408, *p* < 0.001), (β = 63.88, *p* < 0.001) for *Verbal Fluency* and *Trail Making Test*, respectively. The correlation of a history of trauma with the results of the *Trail Making Test* was also statistically significant (β = − 26.01, *p* < 0.041.)

Table 2 shows the results of the univariate and generalized linear model. The Generalized Linear Model (GLM) with poisson loglinear distribution indicated that there was a collectively significant effect between the outcomes and gender, age, education and history of traumatic experience, with a significance of *p* < 0.001 across all the models. Furthermore, the subtype of Pathogenic Possession turned into an independent predictor in all the models: Wisconsin card sorting test perseverative error, *Wisconsin Card Sorting Test* categories achieved and the number of problems solved in the *Tower of London Test* (OR = 3.70, 95% C.I. 2.97–4.61; *p* < 0.001), (OR = 0.57, 95% C.I.0.39–0.84; *p* = 0.004) and (OR = 0.80, 95% C.I. 0.65–0.99; *p* < 0.037) respectively. Participants that had experienced trauma in the past had more statistically significant perseverative errors in the *Wisconsin Card Sorting Test* (OR = 0.81, *p* < 0.020).

**Table 2:** The association between the subscales of *Wisconsin Card Sorting Test* (WCST: Perseverative errors, the number of categories achieved) and *Tower of London* (number of correct moves), subtypes of possession and socio-demographics (gender age and education and risk factors-history of trauma)

Variables		n	WCST (Perseverative errors)				WCST (*the number of categories achieved*)				Tower of London (*number of correct move*)			
			Unadjusted		Adjusted		Unadjusted		Adjusted		Unadjusted		Adjusted	
			Mean (sd)	*p*-Value	β	*p*-Value	Mean (sd)	*p-*Value	β	*p-*Value	Mean (sd)	*p*-Value	β	*p*-Value
Gender	Male	37	7.43 (6.56)	0.022	0.981	0.807	3.08 (0.92)	0.024	1.023	0.877	9.65 (1.95)	0.081	1.039	0.633
	Female	47	11.38 (8.92)				2.60 (0.99)				8.85 (2.14)			
Age	≤ 30 years	44	12.45 (9.06)	0.001	1.190	0.068	3.08 (0.80)	0.016	0.912	0.544	8.68 (2.37)	0.014	1.016	0.853
	≥ 30 years	40	6.55 (5.72)				2.57 (1.09)				9.78 (1.54)			
Subtype	Intermittent dissociative phenomenon	29	18.38 (5.72)	<0.001	2.966	<0.001	1.90 (0.72)	<0.001	0.569	0.004	7.97 (2.46)	0.001	0.802	0.037
	Transitory dissociative phenomenon	55	5.04 (4.79)				3.29 (0.74)				9.85 (1.51)			
Education	University	29	4.76 (4.44)	<0.001	0.939	0.615	3.38 (0.73)	<0.001	1.048	0.772	9.69 (1.37)	0.070	0.960	0.662
	Less than University	55	12.22 (8.53)				2.51 (0.98)				8.95 (2.35)			
History of traumatic experience	Yes	29	6.28 (5.95)	0.002	0.813	0.020	3.14 (0.88)	0.026	1.026	0.863	9.28 (1.60)	0.795	0.972	0.736
	No	55	11.42 (8.65)				2.64 (1.01)				9.16 (2.31)			

## Discussion

On one hand, the prevailing exploration of dissociative phenomenon hinges on whether the phenomenon constitutes ‘culture-bound syndrome’, ‘culture-reactive syndrome’ or ‘atypical’ presentation as depicted in existing psychiatric nomenclature such as the ICD [18] or DSM [19]. These psychiatric nomenclature also refers to dissociative phenomenon as being ‘pathological’ or ‘non-pathological’, something that has generally been debunked in studies emanating from different parts of the world [36, 37]. On the other hand, global ethnographical data have generally supported the existence of two grades of spirit possession labelled ‘central’ vs. ‘peripheral’ possessions [5], ‘possession’ vs. ‘possession trance’ [1] and ‘pathogenic possession’ vs. ‘executive possession’ [4]. In Arab Islamic society, although there are two parallel grades of the dissociative phenomenon, they are not essentially similar to those reported from other parts of the world. In this study, we have operationalized the two types of dissociative phenomena as ‘intermittent dissociative phenomenon’ and transitory dissociative phenomenon’. Pending further scrutiny, the present grade of dissociative phenomena appears to have heuristic value in Oman. This study therefore aimed to examine the neuropsychological profile of those indicating the two types of dissociative phenomenon. The neuropsychological approach attempts to decipher the structure and function of the brain and how an occurrence of possession symptoms may relate to specific cognitive processes and behaviours. Studies exploring the cognitive performance of people with spirit possession are therefore imperative to further delineate grades of the dissociative phenomenon. To our knowledge, our study provides original data and novel insight into this topic.

The present finding suggests a significantly different neuropsychological profile between two grades of spirit possession in Oman. Specifically, this study compared five neurocognitive measures known to be sensitive to executive functioning among the currently defined grades of possession. Compared to the transitory dissociative phenomenon, this study suggested that performance on *Verbal Fluency* and *Trail Making Test* were significantly different among those with the intermittent dissociative phenomenon. Similar differences were noted in *perseverative errors*, the *number of categories achieved* (*Wisconsin Card Sorting Test*) and the *number of correct moves* (*Tower of London*). These neuropsychological domains have often been portrayed to constitute executive functioning. Executive functions have been defined as the cognitive processes that are essential for selecting and successfully monitoring the direction of ongoing behavior with respect to the desired outcome [38]. Some of the well-established cognitive processes under the rubric of executive functioning include attentional control, cognitive inhibition, inhibitory control, working memory, and cognitive flexibility [39]. There is ample evidence to suggest that the integrity of executive functioning strongly correlated with fluid intelligence [40]. Neuroimaging studies have implicated various brain regions including the anterior cingulate cortex and the dorsolateral prefrontal cortex and their reciprocal projections to other brain regions to be critically associated with executive functioning [28, 31, 33, 35].

Cohen [4], Lewis [5] and Bourguignon [1] have dictated discourse on spirit possession by providing contesting paradigms from anthropology to epistemic premise. While these authors have provided typologies that have heuristic value in terms of conceptualizing the complexity of spirit possession, they did not explicitly equate different grades of spirit possession with variation in neuropsychological functioning. Pending further scrutiny, this study suggests that some subtypes of spirit possession are likely to display a peculiar neuropsychological profile, hence exhibiting a greater risk of dysfunction. Available evidence suggests that cognitive impairment tends to persist despite the fluctuation of emotional and behavioural symptoms and neuropsychological impairments are strong determinants of poor quality of life [41, 42]. The present study, therefore, lays groundwork to guide clinicians about which individuals reporting spirit possession would be at greater risk of dysfunction.

Various studies on dissociative phenomenon among Euro-American and non-western populations have suggested disturbances in several neuropsychological domains including those under scrutiny in the present study [43–45]. Similarly, brain areas that have been indicated as being associated with memory integrity and learning, and attention and executive functioning such as hippocampal and prefrontal cortex, have been shown to be dysfunctional in people with dissociative phenomenon [46–49]. Bastos et al. [50] have embarked on a study to examine what they called physiologic correlates of culture-bound dissociation’ in Brazil. In this study, the experimental group (medium group) consisted of the participant who is capable of communicating with the spirit and control group. The medium group endorsed highly on indices of anomalous experience. While the group appeared to differ on indices of catecholamine and monoamine, the difference however did not reach significant levels except for indices of noradrenaline activity. These authors concluded that pathological and nonpathological dissociation is associated with divergent physiological mechanisms. Existing literature suggests that catecholaminergic and monoaminergic neurotransmission tend to invariably effect neuropsychological functioning [51]. In support of such view, preclinical literature on animals with depleted catecholamine, and monoamine tend to perform poorly on dimensions of executive functioning [52].

Lewis [5] has suggested spirit possession to constitute symbolic forms of crying foul for being marginalized. According Lewis [5], downtrodden members of the society attempted to remedy their precarious situation by means of spirit possession. In addition to cultural symbolism, the link between a history of adversity and the development of spirit possession has been suggested in the literature. Neuner et al. [53] have conducted an epidemiological survey among the communities in Northern Uganda whose lives have been massively impacted by the decade of civil conflict. This study indicates that the presence of spirit possession is often precipitated by traumatic events. This view is consistent with the previous suggestion that spirit possession tends to coincide with volatile political situations or times of great socioeconomic tribulation [54, 55]. Within such background, the present study explored the link between having a history of trauma using *the Trauma History Questionnaire* [23, 24] and different grades of possession. The present data suggest that 34.5% of the participants endorsed a history of trauma. The majority (*n* = 44) of those who endorsed a history of trauma belonged to the category of transitory dissociative phenomenon. In contrast, 5 out of 29 indicating intermittent dissociative phenomena endorsed a history of traumatic experiences. Hecker, Braitmayer & van Duijl [56] have synthesized literature on dissociative phenomenon from 1994 until 2014 from medical and psychological search engines. Identifying 54 studies, the authors suggested that the rate of trauma among those with dissociative phenomenon tended to range from 0.5 to 18.6%. While dissociative phenomenon and history of trauma have been widely shown to have a temporal association, there are dissenting views. For example, Moreira-Almeida et al. [57] have compared patients with the dissociative phenomenon from North America to those from South America. This study suggests that Brazilians with dissociative phenomenon endorsed less history of traumatic events. Overall, these contrasting links between trauma and dissociative phenomenon suggest that some grades of the dissociative phenomenon are more prone to be precipitated by traumatic events than others. More studies on this are therefore warranted.

Traumatic experiences have been shown to affect the integrity of the amygdala, hippocampus, and prefrontal cortex [58] and to be strongly associated with heightened cortisol and norepinephrine responses [50]. This would imply that people with a history of trauma would have impaired performance on some of the tasks of the *Trail Making* and *Wisconsin Card Sorting* Tests*,* i.e. *Perseverative errors* as demonstrated previously among war veterans [59, 60]. Contrary to such views, this study suggests the history of traumatic experience to have a complex relationship in dictating neuropsychological profiles. This study suggests that those indicating intermittent dissociative phenomenon appear to perform poorly in the majority of neuropsychological functioning. It is possible that being severely impaired could also render one incapable of recalling certain traumatic memories. In Oman, the occurrence of the intermittent dissociative phenomenon is locally known to suspend one’s identity and selfhood [3]. It is therefore plausible that the widely found association between trauma and the dissociative phenomenon is more apparent among those with less extensive grade of the dissociative phenomenon. More studies on this discourse are therefore warranted.

## Limitations

In traditional Omani society, possession is perceived as being benign and a fundamental part of local cosmology and theology [61]. This means that phenomena akin to possession are likely to remain the prerogative of traditional healers [3]. Only in those circumstances where distress reached irreversible and advanced pathology, and the person was thus deemed ‘sick’, were the individuals likely to be referred to biomedical care. Secondly, this study was not equipped to tease out whether neuropsychological performances were clinically significant or otherwise. This study has established variations in neuropsychological performance among different grades of spirit possession. However, it was beyond the scope of this study to discern whether, for example, poor performance among those with intermittent dissociative phenomenon was the result of possession or the cause. Indeed, it is possible that rather than being part of the ‘illness’, impaired neuropsychological functioning might be a compensatory adaptation to spirit possession. Moreover, this study lacks enough information to determine whether cognitive dysfunction evidenced in the assessments is directly due to a belief of possession or whether lower cognition increases susceptibility to certain categories of possession. It is therefore recommended that future studies compare scores of cognitive functioning among participants of both subtypes of possessions. Another limitation of this study is the utility and specificity of the intermittent dissociative phenomenon and transitory dissociative phenomenon. Since spirit possession is not officially sanctioned in the existing nomenclatures, the presence of spirit possession was solicited by an explanatory model interview [20]. However, those with spirit possession also appeared to fulfil the criteria of dissociative disorders. The question then arises of whether spirit possession is a common dissociative phenomenon and if it should therefore be integrated as part of the spectrum of dissociative disorders. Future studies need to examine whether these spirit possessions and dissociative disorders are neuropsychologically orthogonal entities.

## Conclusions

This study has embarked on the task of comparing neuropsychological functioning in a typology of spirit possession operationalized as intermittent dissociative phenomenon and transitory dissociative phenomenon. The present data suggests that the two grades of dissociative phenomena are significantly different in their neuropsychological profiles. The previously noted ‘trauma hypothesis’ in spirit possession appears to be more common in those fulfilling the label of the transitory dissociative phenomenon. It appears neuropsychological functioning rather than the presence of a history of trauma or otherwise has more bearing on the classification of individuals into the two grades of spirit possession.

## Data Availability

This is a research article and all manipulated data generated or analyzed during this study are included and presented in this published article. The raw datasets used and/or analyzed during the current study available from the corresponding author on reasonable request.

## Abbreviations

CIDI: Composite International Diagnostic Interview
DSM: Diagnostic and Statistical Manual of Mental Disorders
fMRI: Functional Magnetic Resonance Imaging
GLM: Generalized Linear Model
ICD-10: International Statistical Classification of Diseases and Related Health Problems
PET: Positron Emission Tomography scan
SPECT: Single-Photon Emission Computed Tomography
